# Abnormal upregulation of cardiovascular disease biomarker PLA2G7 induced by proinflammatory macrophages in COVID-19 patients

**DOI:** 10.1038/s41598-021-85848-5

**Published:** 2021-03-24

**Authors:** Yang Li, Yongzhong Jiang, Yi Zhang, Naizhe Li, Qiangling Yin, Linlin Liu, Xin Lv, Yan Liu, Aqian Li, Bin Fang, Jiajia Li, Hengping Ye, Gang Yang, Xiaoxian Cui, Yang Liu, Yuanyuan Qu, Chuan Li, Jiandong Li, Dexin Li, Zhongtao Gai, Shiwen Wang, Faxian Zhan, Mifang Liang

**Affiliations:** 1 NHC Key Laboratory of Medical Virology and Viral Diseases, National Institute for Viral Disease Control and Prevention, Chinese Center for Disease Control and Prevention, Beijing, 102206 China; 2 Hubei Provincial Center for Disease Control and Prevention, Wuhan, 430065 China; 3 Qilu Children’s Hospital, Cheeloo College of Medicine, Shandong University and Jinan Children’s Hospital, Jinan, 250022 China; 4 Department of Microbiology, School of Basic Medical Science, Anhui Medical University, Hefei, 230032 China; 5 The Center for Scientific Research of the First Affiliated Hospital of Anhui Medical University, Hefei, 230022 China; 6 Xiantao Center for Disease Control and Prevention, Xiantao, 433000 China; 7 Xiangyang Center for Disease Control and Prevention, Xiangyang, 441000 China; 8 Shanghai Municipal Center for Disease Control and Prevention, Shanghai, 200336 China; 9 CDC-WIV Joint Research Center for Emerging Diseases and Biosafety, Wuhan, 430071 China

**Keywords:** Viral host response, Data mining

## Abstract

High rate of cardiovascular disease (CVD) has been reported among patients with coronavirus disease 2019 (COVID-19). Importantly, CVD, as one of the comorbidities, could also increase the risks of the severity of COVID-19. Here we identified phospholipase A2 group VII (PLA2G7), a well-studied CVD biomarker, as a hub gene in COVID-19 though an integrated hypothesis-free genomic analysis on nasal swabs (n = 486) from patients with COVID-19. PLA2G7 was further found to be predominantly expressed by proinflammatory macrophages in lungs emerging with progression of COVID-19. In the validation stage, RNA level of PLA2G7 was identified in nasal swabs from both COVID-19 and pneumonia patients, other than health individuals. The positive rate of PLA2G7 were correlated with not only viral loads but also severity of pneumonia in non-COVID-19 patients. Serum protein levels of PLA2G7 were found to be elevated and beyond the normal limit in COVID-19 patients, especially among those re-positive patients. We identified and validated PLA2G7, a biomarker for CVD, was abnormally enhanced in COVID-19 at both nucleotide and protein aspects. These findings provided indications into the prevalence of cardiovascular involvements seen in patients with COVID-19. PLA2G7 could be a potential prognostic and therapeutic target in COVID-19.

## Introduction

Severe acute respiratory syndrome coronavirus 2 (SARS-CoV-2) is a novel enveloped RNA betacoronavirus that emerged in December 2019 in Wuhan, China, and is the causative etiology of coronavirus disease 2019 (COVID-19)^1,2^. Typical clinical presentation of COVID-19 was a lung involvement, as evidenced by image tests, with fever, cough and dyspnoea^3,4^. Severe cases often developed acute respiratory distress syndrome (ARDS) or even death^3,4^. The risk factors for increased disease severity in patients with COVID-19 has been reported as older age (e.g., over 50 years old) and the presence of comorbidities, including hypertension, diabetes mellitus, cardiovascular disease etc^5,6^. However, it rapidly became obvious that severe COVID-19 can also occur in younger patients with no pre-existing comorbidities^7^.

On-going data characterizing immunological features in patients with COVID-19 were starting to emerge. Host response to SARS-CoV-2 infection was distinct in comparison with other highly pathogenic coronaviruses and common respiratory viruses in cell lines^8^. Notably, reduced type I interferon activities were observed in both in vitro^8^ and in vivo^9^ data. Delayed production of type I interferon resulted in boosted cytopathic effects (CPE) and increased sensing of SARS-CoV-2 threats promoted the enhanced release of monocyte chemoattractants, such as chemokine ligand 2 (CCL2)^10^, which contributed an influx of monocytes into lungs^7^. Autopsy studies have shown diffuse thickening of the alveolar wall with mononuclear cells and macrophages infiltrating airspaces^11^. Furthermore, postmortems pointed out that multiorgan dysfunction did not map to tissue/cellular distribution of SARS-CoV-2, indicating immune-mediated as opposed to pathogen-mediated organ inflammation contributed to severity in COVID-19 patients^12^.

Hypothesis-free biomarker studies could allow researchers to gain in depth insights into patho-mechanisms underlying COVID-19. Several attempts have been made. Expression of monocyte CD169 (mCD169), also known as sialic acid binding Ig like lectin 1 (SIGLEC1), has been suggested as a biomarker in the early diagnosis of COVID-19^13^. On the other hand, protein-level biomarkers that could predict the severity of COVID-19 patients were identified^14,15^. In particular, the level of lactate dehydrogenase (LDH) in the blood which was used to monitor the tissue damage was highly indicative of COVID-19 mortality with area under curve (AUC) > 0.9^14^. These results suggested the development of additional cell- and/or tissue-type specific host injury markers were needed^16^. It was worthy of noting that cytokine blood RNA level was not always correlated with protein levels in plasma^9^. Interleukin (IL)-6, a key player of hyperinflammation in COVID-19, was not detected at RNA level in blood, contrasting with high protein level^9^, indicating partial cytokines could result from lungs^17^. Herein, we conducted an integrated genomic analysis of samples from respiratory samples to find out such a biomarker of COVID-19 which showed a distinct pattern between RNA and protein level in blood.

## Results

### PLA2G7 was identified as hub gene in SARS-CoV-2 infection

**Figure 1:**
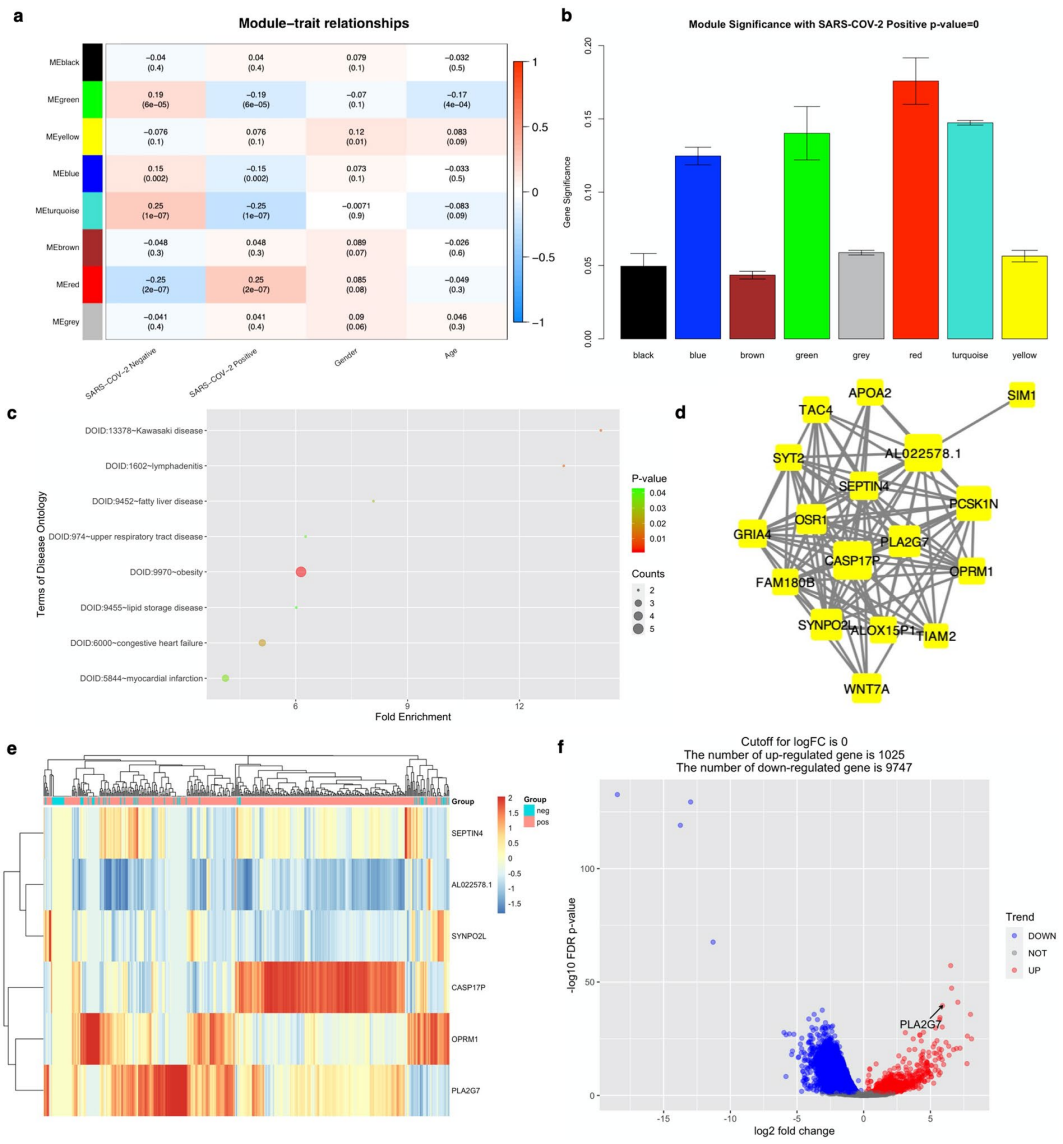
In silico discovery of PLA2G7 as the biomarker of SARS-CoV-2 infection. (**a**) Heatmap of the correlation between module eigengenes and the clinical traits recorded in GSE152075. (**b**) Distribution of average gene significance and errors in the modules associated with positivity of SARS-CoV-2. (**c**) Disease Ontology (DO) enrichment results on genes in red module. (**d**) Visualization of the network connections among the most connected genes in the red module. The size of circles was equal to the log2 fold change. (**e**) Heatmap based on unsupervised clustering of the selected 6 genes after scaling. Each row represented one gene; each column represents one patient. Expression intensity is indicated by color. (**f**) Volcano plot of differentially expressed genes (DEGs) for SARS-CoV-2 infection contrasting positive cases with negative ones in GSE152075.

The clinical records in GSE152075^18^ labelled as ‘not collected’ were removed. Batch-corrected count matrix was normalized by variance Stabilizing Transformation (vst) function in R package DEseq2^19^. Following cluster analysis on the preprocessed expression matrix to remove outliers, in a total of 428 samples out of 484 were remaining (Supplementary Fig. S1). As the scale-free topology fit index failed to reach values above 0.85 (Default Threshold), the soft-thresholding power of 12 was selected (Supplementary Fig. S2). In a total number of 8 modules were identified and constructed by WGCNA^20^ analysis (Fig. 1a; Supplementary Fig. S3). To identify the SARS-CoV-2 infection-related module, the Pearson correlation analysis, which involved calculating the Student asymptotic *P*-values for the correlations, between the module eigengenes (MEs) of each module and clinical traits was performed (Fig. 1b). The red module was the module most relevant to SARS-CoV-2 infection. Genes in red module (n = 60) were listed in Table S1. Importantly, the disease ontology (DO)^21^ analysis on these genes suggested not only upper respiratory tract disease, but also heart disease related terms (e.g., congestive heart failure and myocardial infarction) (Fig. 1c). In addition, the network connections among the most connected genes in the red module were displayed through Cytoscape (version: 3.7.6, http://cytoscape.org)^22^ (Fig. 1d).

The genes in the red module were selected for feature reduction analysis. In a total of 6 genes were selected based on a gene significance threshold of 0.8 and a module membership significance of 0.25 (Fig. 1e; Supplementary Fig. S4). Next, single-feature gene selection based on XGBoost^23^ was carried out. First of all, all the samples were randomly assigned at a 7:3 ratio to a training set (299 samples) and a test set (129 samples). The python packages “XGBoost”^23^ and “scikit-learn”^24^ were used for classification. Second, to obtain the best XGBoost model parameter combination (eta, gamma, max_depth, Min_child_weight, Subsample, and n_ Colsample_bytree) with the highest classification accuracy, five-fold cross-validation and grid search were applied to the training set. Finally, the highest accuracy of classification was 0.876 which could be achieved through a single gene, phospholipase A2 group VII (PLA2G7) (Supplementary Fig. S5). Moreover, the AUC score by R package “pROC”^25^ in the training and test sets on this single gene was 0.850 and 0.820, respectively (Supplementary Fig. S6). It should be noted that PLA2G7 was significantly upregulated in SARS-CoV-2 positive group (logFC = 5.903, q-value = 2.68e-40) (Fig. 1f).

### PLA2G7 was induced by proinflammatory macrophages emerging along with progression of COVID-19

**Figure 2:**
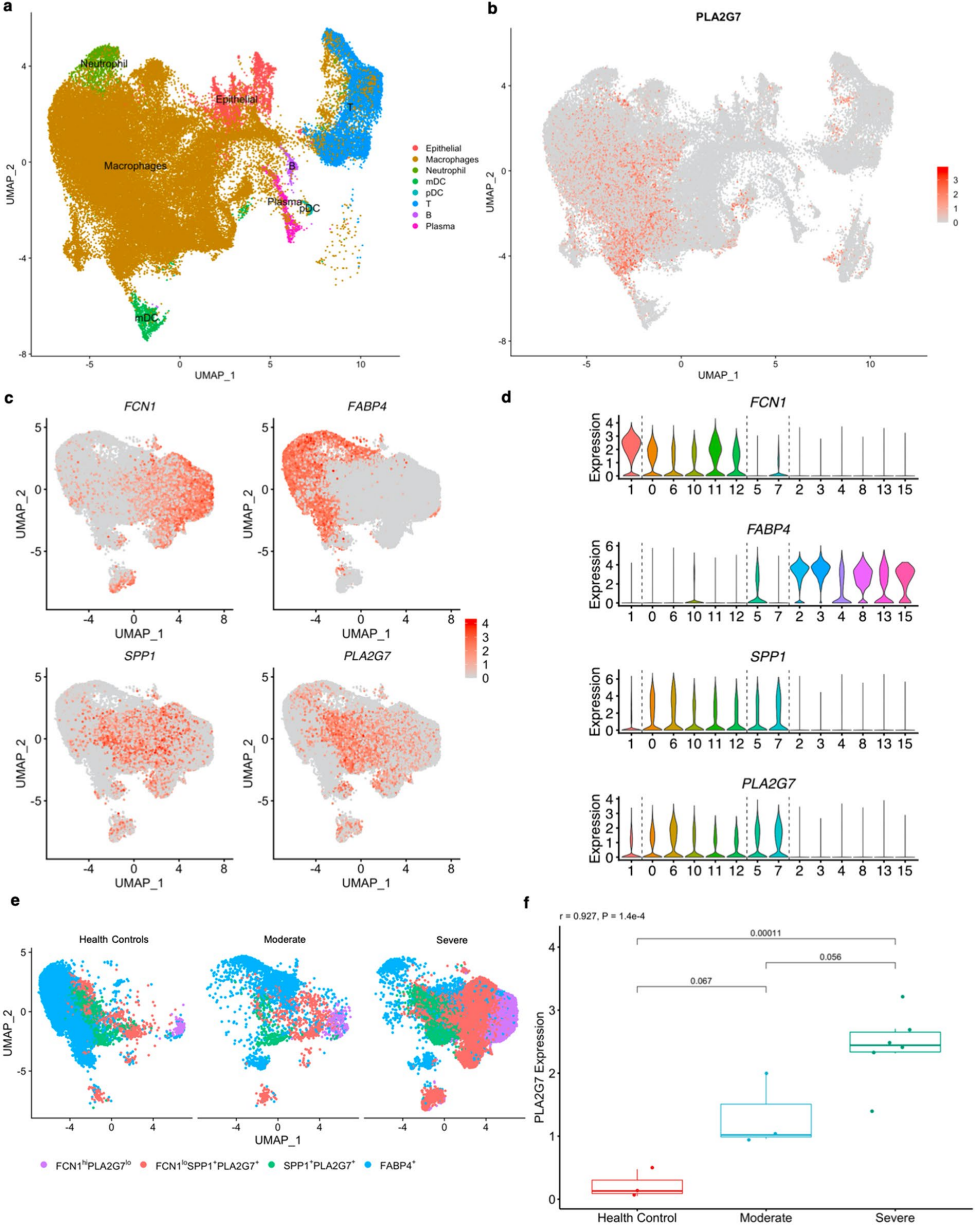
PLA2G7 was induced by proinflammatory macrophages emerging along with progression of COVID-19. (**a**) UMAP presentation of major cell types in bronchoalveolar lavage fluids (BALFs) from patients with COVID-19 in GSE145926. (**b**) UMAP plot showing the expression of PLA2G7 across the immune cells. (**c**) UMAP maps showing the expression of PLA2G7, along with the markers (SPP1, FCN1 and FABP4) in re-integrated macrophages. (**d**) Macrophages were classified into 4 groups based on the expression of FCN1, FABP4, SPP1 and PLA2G7, indicating by dashed lines. (**e**) UMAP projection of four macrophage groups among the health controls, patients with moderate and severe COVID-19, indicating the dynamic changes of macrophages in different patients. (**f**) Expression of PLA2G7 was correlated with progression of COVID-19. UMAP, Uniform Manifold Approximation and Projection.

Clustering analysis on scRNA-seq data (GSE145926) showed 27 distinct clusters composed of macrophages, neutrophils, myeloid dendritic cells (mDCs), plasmacytoid dendritic cells (pDCs), natural killer (NK) cells, T cells, B cells, plasma cells and epithelial cells (Fig. 2a; Supplementary Fig. S7–8). The gene expression analysis showed PLA2G7 was expressed principally by macrophages (Fig. 2a,b). To further understand the origin of PLA2G7, the 17 macrophages subclusters were re-integrated and re-clustered (Supplementary Fig. S9). Thereafter, the macrophages were grouped based on the expression profile of recent markers (Ficolin 1 [FCN1], Secreted Phosphoprotein 1 [SPP1] and Fatty Acid Binding Protein 4 [FABP4])^26,27^ and present PLA2G7 (Fig. 2c,d). Four heterogeneous subgroups of macrophages, including groups of FCN1^hi^PLA2G7^lo^, FCN1^lo^SPP1^+^PLA2G7^+^, SPP1^+^PLA2G7^+^ and FABP4^+^, those were identified as previously described (Fig. 2d)^26^. The expression profile of PLA2G7 exhibited a distant pattern to FABP4, but a close pattern to SPP1 (Fig. 2c,d), which played an important role in idiopathic pulmonary fibrosis^27^. Macrophages with FABP4, known as the alveolar macrophages, were reduced while macrophages with FCN1 and SPP1, considered as the monocyte-derived proinflammatory macrophages, were strikingly increased in COVID-19 patients (Fig. 2e). Furthermore, strong correlation between expression of PLA2G7 and the severity of COVID-19 was found (r = 0.927, *P* value = 1.4e − 4) (Fig. 2f). On the whole, PLA2G7 was predominantly expressed by monocyte-derived proinflammatory macrophages emerging along with progression of COVID-19.

### PLA2G7 was detected in a retrospective collection of nasal swabs from both COVID-19 and pneumonia patients

**Figure 3:**
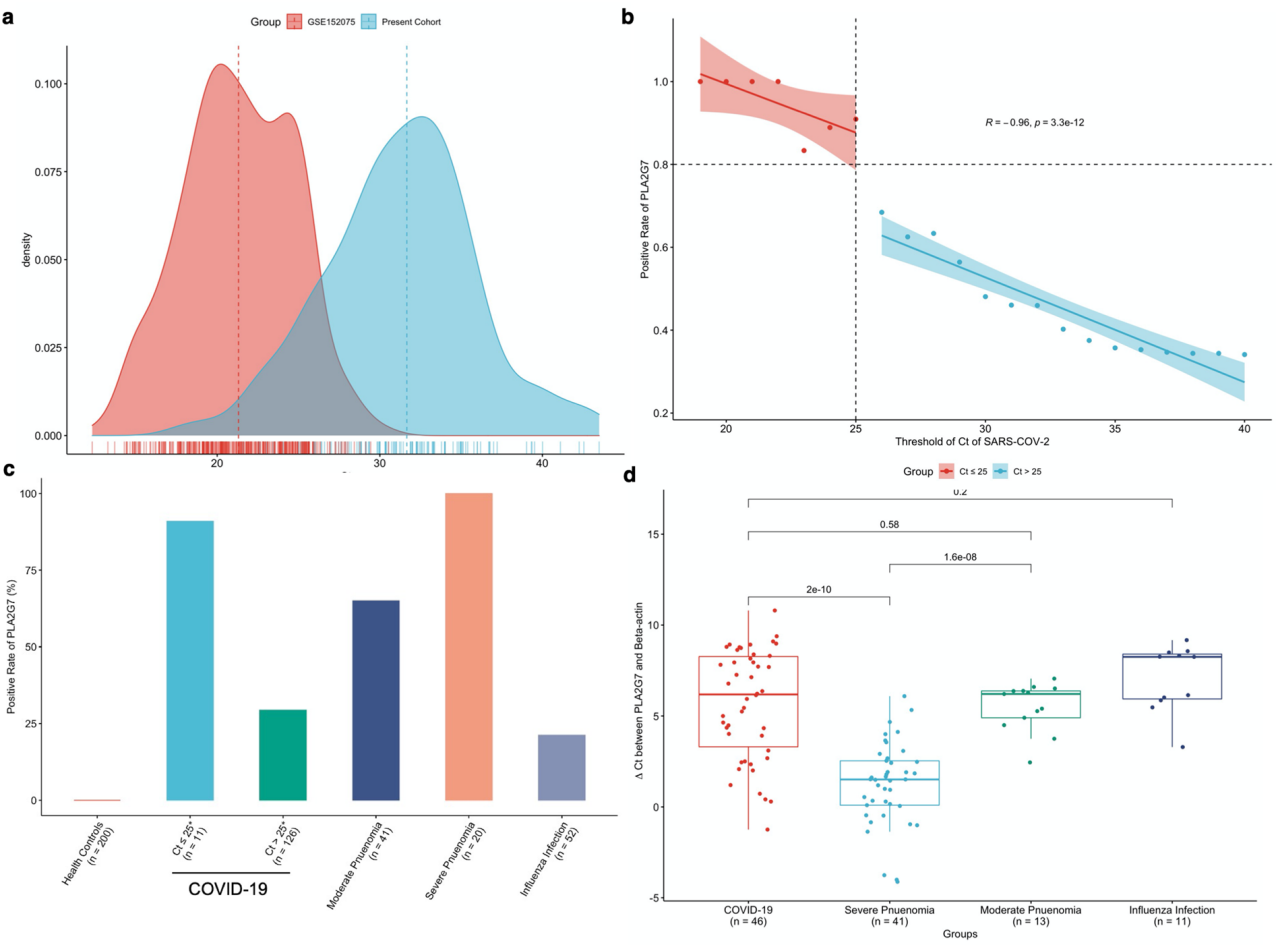
PLA2G7 was detected in a retrospective collection of nasal swabs from both COVID-19 and pneumonia patients. (**a**) Density plot of SARS-CoV-2 Ct values distribution in GSE152075 and present cohorts. (**b**) Relationship between the positive rate of PLA2G7 changed along with the Ct values of SARS-CoV-2. (**c**) Positive rates of PLA2G7 in patients’ group. **(d)** Comparison on the expression of PLA2G7 across samples was based on the ∆Ct which was defined as differences between Ct_(PLA2G7)_ and Ct_(ß-actin)_.

In a total of 447 nasal swabs were collected and subject to SARS-CoV-2 targeted qRT-PCR. The 447 nasal swabs were grouped as: Health controls (n = 200), COVID-19 group (n = 134), Influenza infection (n = 52), Severe pneumonia (n = 41) and Moderate pneumonia (n = 20). It should be noted the median (interquartile range [IQR]) of Ct of COVID-19 group in present study were relatively higher than that in public dataset GSE152075 (SARS-CoV-2 Ct: 31.66 [28.62–34.00] vs. 21.31 [19.09–24.01]) (Fig. 3a). To match the records in GSE152075, the COVID-19 group were divided into two groups with Ct value ≤ 25 and > 25. Thereafter, we next explored the relationship between positive rates of PLA2G7 and viral load of SARS-CoV-2 in COVID-19 group. The positive rates of PLA2G7 were correlated with Ct of SARS-CoV-2 (r =  − 0.96, *P* value = 3.3e − 12) (Fig. 3b), indicated that the positive rates of PLA2G7 was positively correlated with SRAS-CoV-2 viral load. It was worth of noting more than 80% PLA2G7 positive rates were observed in COVID-19 patients with Ct of SARS-CoV-2 ≤ 25 (Fig. 3b,c), which showed comparable diagnostic performance in GSE152075 as shown in Fig. 1e.

The positive rates of PLA2G7 in all different groups were shown in Fig. 3c. There were no PLA2G7 positive cases in health controls. Among the 134 SARS-CoV-2 positive cases, 47 (35.1%) were PLA2G7 positive. It was worth of noting more than 90% PLA2G7 positive rates were observed in COVID-19 patients with Ct of SARS-CoV-2 ≤ 25 while 29.37% were seen in the group with Ct of SARS-CoV-2 > 25 (Fig. 3b,c). Surprisingly, a similar high detection rate of PLA2G7 was seen in patients with pneumonia (SRAS-CoV-2 negative). Notably, 100.00% of PLA2G7 positive rates were seen in group of severe pneumonia while 65.00% were noticed in moderate pneumonia patients. Conversely, only about 20.00% PLA2G7 positive rates were observed in patients with influenza infection. To compare the expression of PLA2G7 across samples, ΔCt which was defined as differences between Ct_(PLA2G7)_ and Ct_(ß-actin)_ was applied. The ΔCt of PLA2G7 in group of SARS-CoV-2 shared a similar pattern to that in group of moderate pneumonia. Notably, ΔCt of PLA2G7 in group with severe pneumonia were lower than that in group with moderate pneumonia (*P* value = 1.6e − 8), indicating PLA2G7 could be associated with the severity of pneumonia (Fig. 3d).

### Abnormal serum protein levels of PLA2G7 were examined in COVID-19 patients

**Figure 4:**
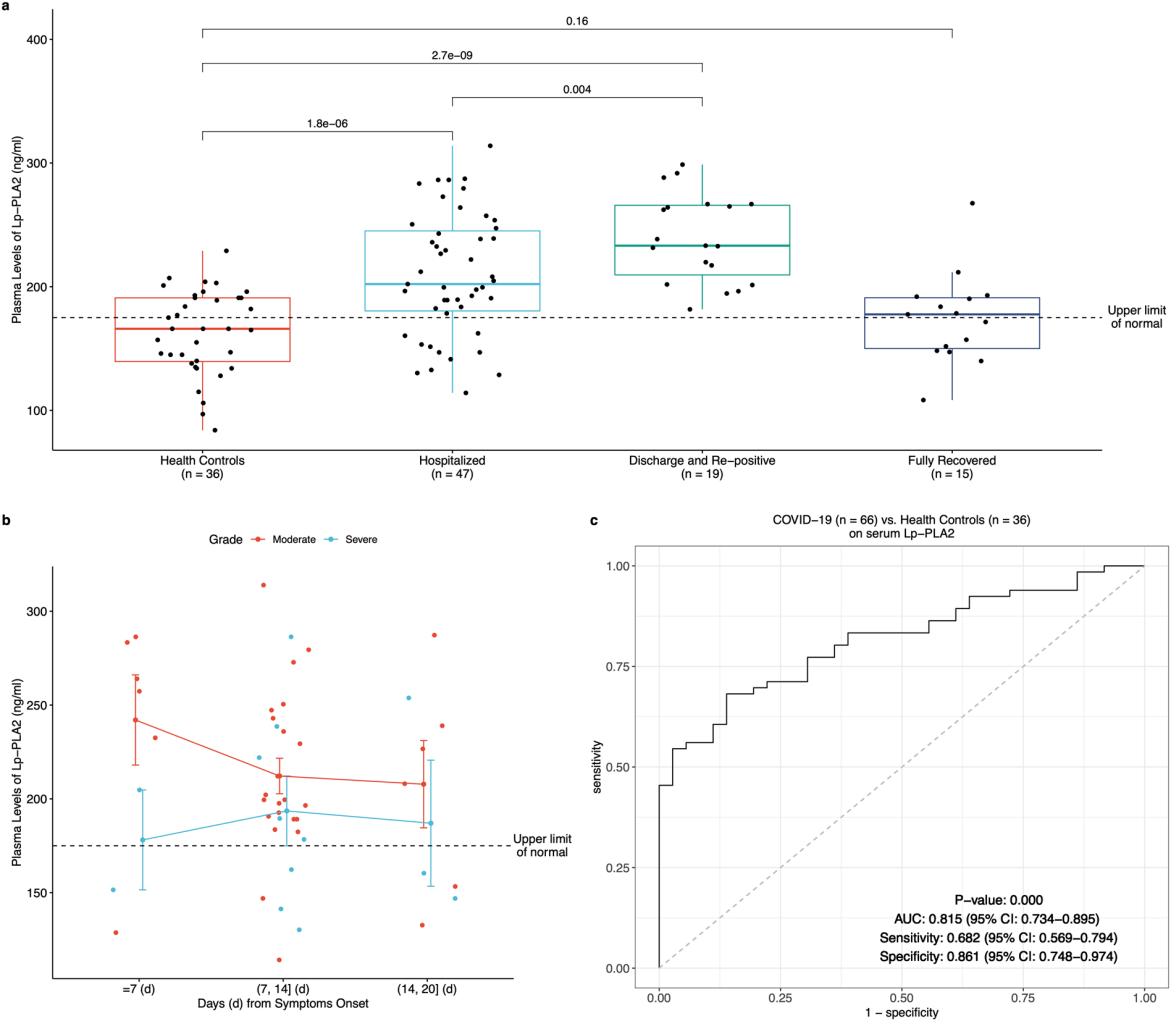
Abnormal plasma levels of Lp-PLA_2_ in patients with COVID-19. (**a**) plasma levels of Lp-PLA_2_ in four groups of patients with COVID-19. (**b**) Plasma levels of Lp-PLA_2_ in hospitalization group along with days (**d**) from the initial symptoms onset. (**c**) Evaluation of classification performance of plasma levels of Lp-PLA_2_ between COVID-19 patients and health controls.

To understand the plasma levels of PLA2G7 which was also known as lipoprotein-associated phospholipase A_2_ (Lp-PLA_2_) in plasma^28^, a total of 117 serum samples (36 health controls, 81 COVID-19 patients) were collected. Among the 81 COVID-19 patients, 47 serum samples were collected at various time points from patients during the hospitalization (Hospitalization Group), 19 were from the patients who had been discharged and then re-positive but asymptomatic (Re-positive Group), and 15 were from fully recovered individuals (Recovered Group). Compared with the health controls, protein levels of Lp-PLA_2_ were higher in hospitalization and re-positive patients (Fig. 4a). It was worth of noting that the hospitalization group showed significantly less levels of Lp-PLA_2_ than that in re-positive group (*P* value = 0.004). Thereafter, we sought to explore the trend of Lp-PLA_2_ along with time among hospitalization group. The overall trend of Lp-PLA_2_ in hospitalization group was reduced along with the days from initial symptoms onset (Fig. 4b). To our surprise, higher plasma levels of Lp-PLA_2_ was in moderate than in severe patients (Fig. 4b). In addition, the serum levels of Lp-PLA_2_ showed discriminating power between patients with COVID-19 and health individuals with AUC of 0.815 (95%CI 0.734–0.895) (Fig. 4c).

## Discussion

Given that the morbidity and mortality seen in COVID-19, a better understanding of the immunological underpinnings seen in patients infected with SARS-CoV-2 is necessary to better identify biomarkers, especially these represented the diagnostic, prognostic and therapeutic targets. It has been suggested that cytokines blood RNA levels could not always be correlated with the protein plasma level^9^, indicating partial cytokines might be from the damaged lungs. Notably, cytokines as IL-6, tumor necrosis factor (TNF) were found to be expressed by lung macrophages from patients with severe COVID-19 infection^26^. Based on the combination of bulk transcriptomic data from nasal swabs (n = 484) and scRNA-seq data from BALF of COVID-19 patients, proinflammatory monocytes-driven macrophages specific PLA2G7 was identified as an import role in SARS-CoV-2 infection and it could provide alternative insights into patho-mechanisms underlying COVID-19.

In the validation stage, expression patterns of PLA2G7 in more than 400 nasal samples were examined by qRT-PCR. Comparing to the diagnostic performance of PLA2G7 in public dataset (Supplementary Fig. S6), the positive rate of PLA2G7 in SARS-CoV-2 positive samples was relatively low (Fig. 3). This discrepancy might be due to the relatively low viral load in present cohort (Fig. 3a). It was noted that positive rate of PLA2G7 in SARS-CoV-2 positive samples were over 0.8 when the Ct of SARS-CoV-2 was lower than 25 (Fig. 3b). As proinflammatory macrophages in lungs played central roles in pneumonia^29^, we postulated that PLA2G7 could be detected in not only patients with COVID-19, but also patients with pneumonia. The positive rate of PLA2G7 in nasal swabs from donors with pneumonia was higher than that in health controls and patients suffered seasonal influenza infection (Fig. 3c). It was worth of noting that 100% positive rate of PLA2G7 were observed in severe pneumonia. In addition, the ΔCt of PLA2G7 were lower in severe pneumonia than in moderate pneumonia (*P* value = 1.6e − 8). This was consistent with our findings that expression of PLA2G7 was correlated with severity of COVID-19 (Fig. 2f), suggesting PLA2G7 could be also used to monitor the progression of pneumonia. On-going epidemiological evidence substantiated the correlation between pneumonia and subsequent CVDs^30^. Up to 30% of patients hospitalized for Community-acquired pneumonia (CAP) developed CVDs^31^. Moreover, pneumonia has been suggested as a risk factor of CVDs^32^. However, the patho-mechanisms by which pneumonia may trigger or promote subsequent CVDs still remain unclear. The expression profile of PLA2G7 was observed in patients with pneumonia could help to understand patho-mechanisms underlying pneumonia and CVDs.

The abnormal levels of plasma protein of Lp-PLA_2_ have been seen in patients with COVID-19, especially the non-hospitalization patients (Fig. 4). First of all, the aberrant plasma levels of Lp-PLA_2_ were observed in patients with COVID-19 from not only hospitalization group, but also re-positive group (Fig. 4a). Strikingly, the plasma level of Lp-PLA_2_ in hospitalization group was significantly lower than that in re-positive group (*P* value = 0.004) (Fig. 4a). It was necessary to point out all the patients in re-positive group were asymptomatic. Therefore, the re-positive patients who had great potential risks on CVDs might, however, receive limited medical attention on CVDs. In addition, although there was no significant difference (*P* value = 0.16) in the plasma level of Lp-PLA_2_ between fully recovered patients with COVID-19 and health controls, it was worried to state that the mass of Lp-PLA_2_ in more than half (53.3%) of recovered patients (n = 15) was beyond the upper limit of normal (Fig. 4a). Coincidentally, a recent study showed 78% of recovered patients with COVID-19, including illness ranging from asymptomatic to moderate symptoms, suffered issues association with the heart^33^. Secondly, although the Lp-PLA_2_ was reduced along with time in hospitalized patients with COVID-19, it should be noted that the plasma levels of Lp-PLA_2_ in these patients were beyond the upper limit of normal, especially the moderate patients (Fig. 4b). According to National Health Commission of China (NHC), some of the patients (later COVID-19 confirmed cases) first went to see a doctor because of cardiovascular symptoms, rather than respiratory symptoms^34^. Even without symptoms and signs of interstitial pneumonia, cardiac involvement as a complication associated with COVID-19 has been reported^35^. Compare to severe group, the moderate patients who always presented fewer and/or lighter symptoms of CVDs leaded to limited medical care on CVDs. This might initiate follow-up comorbidities with heart in these patients.

In addition to CVDs, abnormal enhancement of sera PLA2G7 leaded to impaired arachidonic acid (AA)-mediated metabolism. PLA2G7 is a member of the arachidonic acid (AA) releasing PLA_2_ family. AA could be converted into various types of PGs by cyclooxygenase-1 (COX-1) and COX-2. Among them, prostaglandin E_2_ (PGE_2_) by COX-2 was one of the most active molecules. Notably, it could be speculated that COX-2 could be enhanced by SARS-CoV-2 as both nucleocapsid protein (N) and spike glycoprotein (S) of SARS-CoV could attach to the promoter of COX-2 gene, which resulted in boosting production of COX-2 in a dose-dependent manner^36^. Accumulation of PGE_2_ caused by overexpressed COX-2 inhibited the type I IFN activity and production^37^. For example, microsomal prostaglandin E synthase-1 (mPGES-1) gene deleted macrophages exhibited an early antiviral activity compared to wildtype macrophages, resulted in boosted type I IFN and decreased viral load, which was drastically inhibited by addition of exogenous PGE_2_^37^. Although there was no direct proof that targeted inhibition of PLA2G7 could be helpful in COVID-19, it has been stated that inhibition of cytosolic phospholipase A2α (cPLA2α) significantly reduced viral replication of Human Coronavirus 229E (HCoV-229E) and Middle East respiratory syndrome coronavirus (MERS-CoV) infected cells^38^. Importantly, improved levels of PGE_2_ were observed in patients with COVID-19^39^. Given that the releasing factor and products of AA were abnormally elevated, we speculate that irregular levels of AA could be observed in patients with COVID-19.

Nevertheless, our study has certain limitations. The PLA2G7 was not validated in nasal swabs from the patients with severe COVID-19. In addition, the plasma levels of PLA2G7 in pneumonia patients were also not tested. Due to limited information of clinical traits, confounding factors, such as pre-existing CVDs, which could increase the likelihood of PLA2G7 positive case, were not controlled. The in-house qRT-PCR assay of PLA2G7 was not optimized, leading to inadequate diagnostic performance. Most of the samples were retrospectively collected in Wuhan, China. It has been almost three months passed since all the samples were collected. The freshness of samples could also undermine the performance of the in-house PLA2G7 targeted qRT-PCR and commercial Lp-PLA_2_ ELISA toolkit.

Taken together, PLA2G7 which was revealed as a biomarker in COVID-19 by integrated hypothesis-free single biomarker analysis, provided alternative insights into prevalence of cardiovascular involvements seen in COVID-19 patients and patho-mechanism underlying COVID-19. Although the long-term health effects of role of PLA2G7 in COVID-19 cannot yet be determined, on-going epidemiological evidence substantiated heart issues were occurred after COVID-19. Thus, our findings may provide an indication of potentially considerable burden of CVDs in large and growing parts of the population and urgently require confirmation in a larger cohort.

## Materials and methods

### Data collection

In brief, data were obtained from the Gene Expression Omnibus (GEO) database (http://www.ncbi.nlm.nih.gov/geo/) in July 2020 using the keyword “SARS-CoV-2”. The following exclusion criteria were applied to the expression profiling by high throughput sequencing: (1) concerned only cell model; (2) no or insufficient clinical data; and (3) used non-baseline (“healthy”) controls. After review, GSE152075, which contained 484 samples of nasal swabs, was selected for hub gene discovery. Among them, in a total of 430 subjects were positive with SARS-CoV-2 infections while 54 were negative. According to batch information, the batch effect hidden in the raw count matrix of GSE152075 was removing by Combat_seq in the R package sva^40^. Next, GSE145926 which included single-cell RNA sequencing (scRNA-seq) data on bronchoalveolar lavage fluid (BALF) cells from three patients with moderate COVID-19, six patients with severe/critical infection and three healthy controls were also retrieved^26^.

### Differentially expressed genes screening

When the batch-corrected count matrix of GSE152075 was compared in SARS-CoV-2 positive cases with negative cases, DESeq2^19^ was applied for differential expressed genes (DEGs). Thereafter, correction for multiple testing was addressed by controlling the false discovery rate (FDR) using the Benjamini and Hochberg (B.H.) method for both above. Criteria for DEGs were an absolute log_2_ fold change (Log_2_FC) of 0 and the FDR-adjusted *P* value of < 0.05.

### Co-expression network construction

A co-expression network was constructed using the normalized GSE152075 data by the weighted correlation network analysis (WGCNA) in R^20^. The WGCNA algorithm was described in detail previously^41^. First of all, quality assessment of GSE152075 samples was conducted using the cluster method to remove outliners. Secondly, we established the correlation matrix and determined the soft threshold power by analyzing the scale-free network topology with the type of network set to signed. Thereafter, the topological overlap matrix (TOM) was constructed with the minimum number of genes in each module was 30 and the threshold for cut height was set to 0.25 to merge possible similar modules^20^. Based on the trait data of the groups, we calculated each module's *P*-value using a Student t-test gene significance (GS).

To explore the target module, Pearson's correlation analysis was used to examine the association between module eigengenes (MEs) and SARS-CoV-2 positivity. To identify candidate hub genes, we chose the module with the highest correlation coefficient with the SARS-CoV-2 positive, and the genes that had the maximum absolute value of the Pearson's correlation in the module.

### Identification of modules related to SARS-CoV-2 infection

For a given module, the expression profile was summarized into a single characteristic expression profile, designated module eigengenes (MEs). MEs were considered as the first principal component in the principal component analysis (PCA). Thereafter, a Pearson correlation analysis, calculating the student asymptotic *P* values for the correlations, between MEs and clinical traits (SARS-CoV-2 negative, SARS-CoV-2 positive, Gender and Age) was conducted.

### Disease ontology (DO) analyses

To understand the functions of enriched genes in interesting modules, DO^21^ analyses were performed using clusterProfiler^42^, identifying significant results based on a *P*-value ≤ 0.05 and gene counts ≥ 2.

### Candidate hub gene selection

First, the module that was most highly correlated with influenza infection was selected. Hub genes in the module were determined by both gene significance and module membership. Thereafter, hub genes common to both networks were chosen. Finally, a single hub gene was selected using XGBoost with recursive feature elimination with cross-validation (RFECV)^23,24^.

### Sample integration, dimensionality reduction and clustering on scRNA-data

Count matrixes from 10× CellRanger hdf5 in GSE145926 were obtained. The following criteria were then employed to filter low-quality cells: gene number between 200 and 6000, unique molecular identifier (UMI) count > 1000 and mitochondrial gene percentage < 0.1. Thereafter, the gene-cell matrixes of all samples were integrated with Seurat (version: 3) to remove batch effects across different donors. Next, the integrated gene-cell matrix was normalized using ‘LogNormalize’ methods in Seurat^43^ with default parameters. The top 2000 variable genes were then identified using the ‘vst’ method in Seurat FindVariableFeatures function. Variables ‘nCount_RNA’ and ‘percent.mito’ were regressed out in the scaling step. PCA was performed using the top 2000 variable genes. Then UMAP and tSNE was performed on the top 50 principal components (PCs) for visualizing the cells. Graph-based clustering was performed on the PCA-reduced data for clustering analysis with Seurat v3. The resolution was set to 1.2 to obtain a finer result^26^. Briefly, the first 50 PCs of the integrated gene-cell matrix were used to construct a shared nearest-neighbor graph (SNN; FindNeighbors() in Seurat) and this SNN was used to cluster the dataset (FindClusters()) using a graph-based modularity-optimization algorithm of the Louvain method for community detection.

### Macrophages re-integration

Macrophages of all samples were re-integrated using the first 50 dimensions of canonical correlation analysis and PCA with the parameter k.filter was set to 115. In the clustering step, parameter resolution was set to 0.8.

### Ethics statement

The research protocol was approved by the human bioethics committee of the Chinese Center for Disease Control and Prevention, and all participants provided written informed consent. The study conformed to the *Declaration of Helsinki*.

### Retrospective collection of clinical samples and case definition

We enrolled nasal swabs from donors with health condition and diagnosis of COVID-19, influenza infection and pneumonia. In a total of 447 nasal swabs were collected. Among these samples, 200 were from health individuals; 134 moderate pneumonia cases of confirmed diagnosis of COVID-19 (SARS-CoV-2 positive) and 20 suspected COVID-19 cases (SARS-CoV-2 negative) with moderate pneumonia were collected from government authorized hospitals for COVID-19 in Wuhan during the outbreak of COVID-19 (January 2020 to April 2020); 52 cases with H1N1 infection were also included in Wuhan from October to November, 2019 while 41 cases from hospitalized children who were diagnosed as severe pneumonia (SARS-Cov2 negative) were collected from Qilu Children's Hospital, Jinan, Shandong in February, 2020. For the 117 serum samples, in a number of 81 serum samples from patients with COVID-19 and 36 from health donors were also collected from government authorized hospitals for COVID-19 in Wuhan during the outbreak of COVID-19. Among the 81 patients with COVID-19, 47 serum samples were collected at various time points from patients during the hospitalization, 19 were from the patients who had been discharged and then re-positive but asymptomatic, and 15 were from fully recovered individuals. There were 34 moderate cases, 8 severe cases and 5 critical cases in the 47 serum samples from hospitalized patients. Disease severity of the patients with COVID-19 were defined as mild, moderate, severe and critical based on the Interim Guidance for Novel Coronavirus Pneumonia (Trial Implementation of Seven Edition) by the National Health Commission of China issued on 3 March 2020 (http://www.nhc.gov.cn/yzygj/).

### qRT-PCR assay for SARS-CoV-2 and PLA2G7

All the nasal swabs were sent to BSL-3 lab in Hubei CDC with the standard operational procedure (SOP) of the biosafety laboratory to re-test the SARS-CoV-2 RNA before detection of PLA2G7. Total nucleic acid was extracted using the QIAamp viral RNA mini kit (Qiagen) and SARS-CoV-2 targeted qRT-PCR was performed using a China Food and Drug Administration-approved commercial kit specific for SARS-CoV-2 detection (Da An Gene). This kit is based on one-step TaqMan qRT-PCR technique, and ORF1ab and N genes are selected as amplification target regions. Briefly, the 25-µL reaction mixture comprised 17 µL of PCR reaction Solution A, 3 µL of PCR reaction Solution B, and 5 µL of RNA extracts. The cycling conditions were as follows: 50 °C for 15 min, 95 °C for 15 min, followed by 45 cycles for 15 s at 94 °C and 45 s at 55 °C. Each qRT–PCR assay provided a threshold cycle (Ct) value, indicating the number of cycles surpassing the threshold for a positive test. The Ct value for a positive specimen was set at 40 cycles.

To study PLA2G7 transcripts expression, the primers and TaqMan probes selected from the Universal ProbeLibrary Assay Design Center (Roche Diagnostics) were applied. In brief, we quantitated the Ct values of PLA2G7 and the ß-actin gene by AgPath-ID One-Step RT-PCR Reagents (Applied Biosystems) using a CFX96 Real-Time PCR Detection System (Bio-Rad). Cycling conditions were 45 °C for 10 min and 95 °C for 10 min, followed by 40 cycles of 95 °C for 15 s, 58 °C for 45 s. At least three replicates were studied for each sample. Comparison on the expression of PLA2G7 across samples was based on the ΔCt which was defined as differences between Ct_(PLA2G7)_ and Ct_(ß-actin)_.

### Measurement of plasma levels of PLA2G7

The plasma levels of PLA2G7 were detected by enzyme linked immunosorbent assay (ELISA) according to the instruction (Hotgen, registration no. 20192400366). In brief, serum was heat-inactivated at 56℃ for 45 min. Each sample (20 µL) and standard were added to a 96-well plate. A total of 100 μl of enzyme-labeled reagent was added to all wells (except the blank well) and incubated at 37 °C for 1 h. The plate was washed 5 times before chromogenic reagents A and B (50 μl of each) were mixed and added to each well. The plate was incubated in the dark at 37 °C for 10 min before 50 μl stop solution was added to each well to terminate the color reaction. The optical density (OD) 450 nm values in each well were determined by a microplate reader. The levels of PLA2G7 were then calculated according to the standard curve. The upper limit of normal level of PLA2G7 were 175 ng/ml.

### Statistical analysis

R (version 4.0.2) was used for most analyses, with hub gene selection being performed using XGBoost and Scikit in Python (version 3.6). Differences of median percentage among in group were compared using a Student’s t-test (two-sided, unadjusted for multiple comparisons) with R ggpubr v.0.2.5. *P* value ≤ 0.05 was considered statistically significant.

## Supplementary Information

Supplementary Information

## Data Availability

The microarray datasets GSE152075 and GSE145926 for this study can be found in the Gene Expression Omnibus (GEO) database hosted by the National Center for Biotechnology Information of the US National Institutes of Health (https://www.ncbi.nlm.nih.gov/geo/). All data are available in the main text or the supplementary materials.
