# Supplementary material for: Abnormal upregulation of cardiovascular disease biomarker PLA2G7 induced by proinflammatory macrophages in COVID-19 patients
Source: Sci Rep. 2021 Mar 24;11:6811. doi: 10.1038/s41598-021-85848-5 (PMC7990942; doi:10.1038/s41598-021-85848-5)
Supplement: Supplementary file 1 — Supplementary Information [file 41598_2021_85848_MOESM1_ESM.docx]

Supplementary Materials

Abnormal Upregulation of Cardiovascular Disease Biomarker PLA2G7 Induced by Proinflammatory Macrophages in COVID-19 patients

Li et.al


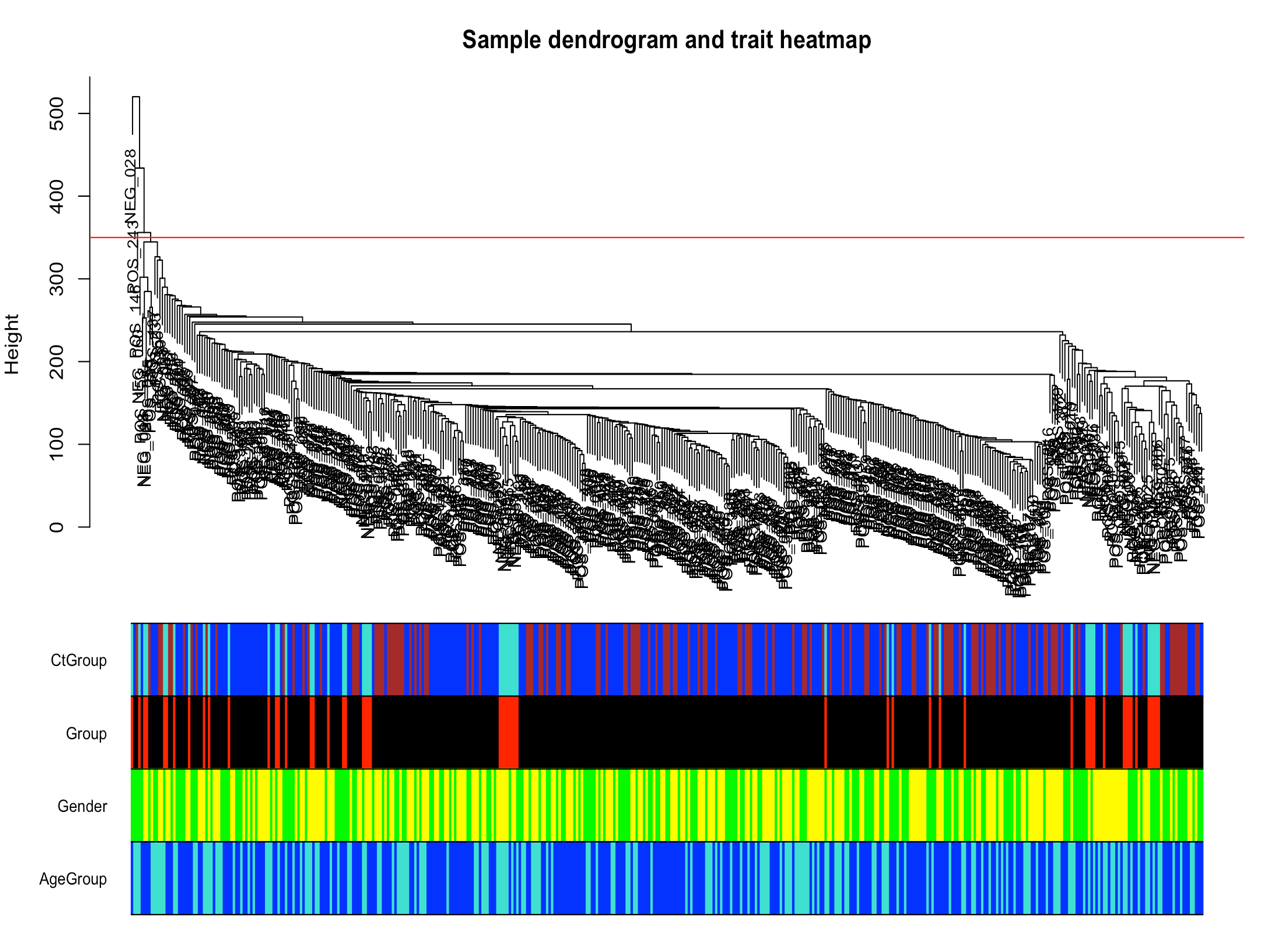


Supplementary Fig. S1. Samples clustering to detect outliers (GSE152075): sample dendrogram and trait indicator with cut height setting to 350. CtGroup was defined according to whether the Ct was larger than 25. AgeGroup was defined according to whether the Age was larger than 50.


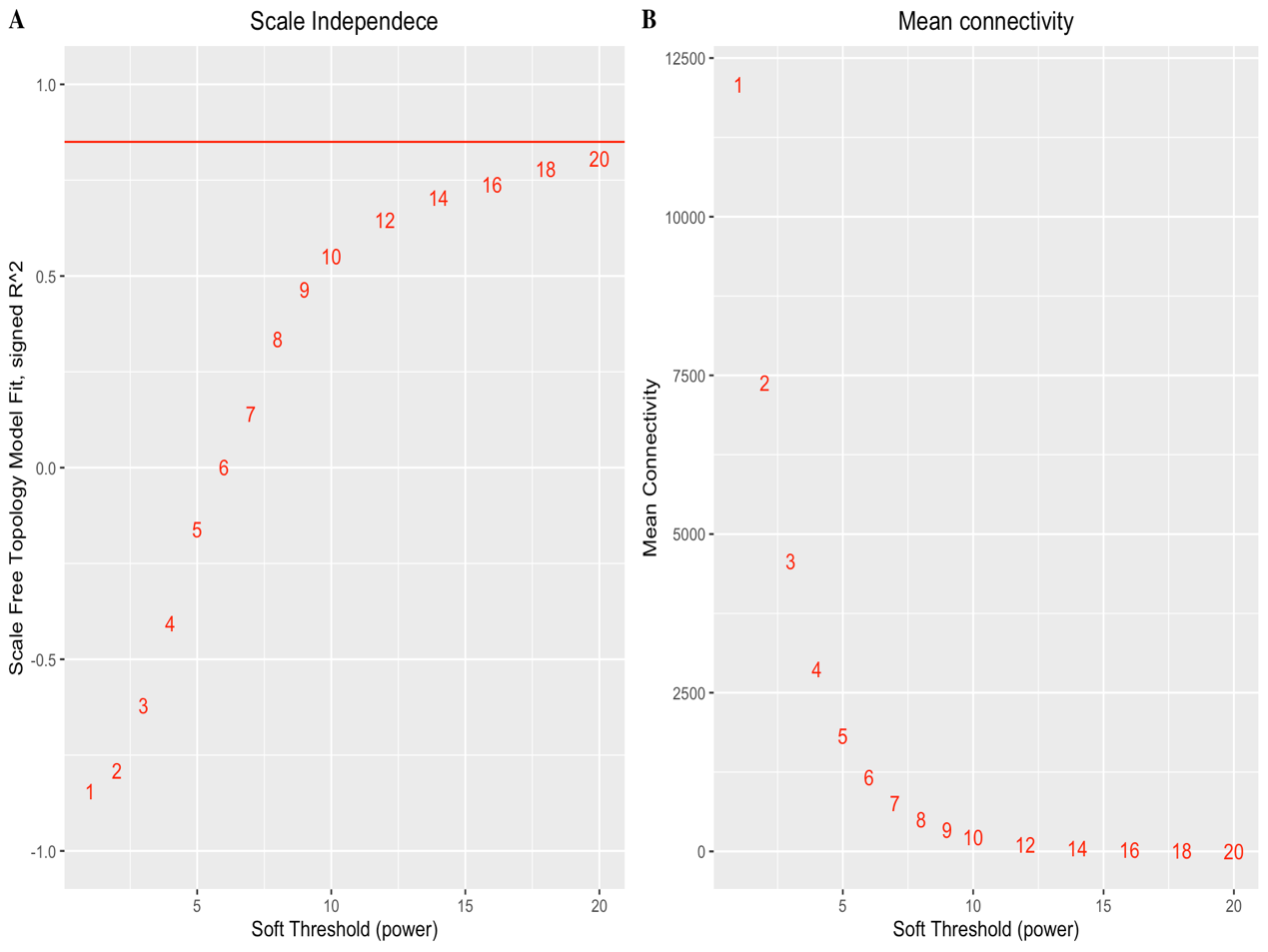


Supplementary Fig. S2. Analysis of the scale-free ﬁt index with a threshold of 0.85 (A) and mean connectivity (B) for various soft-thresholding power values.


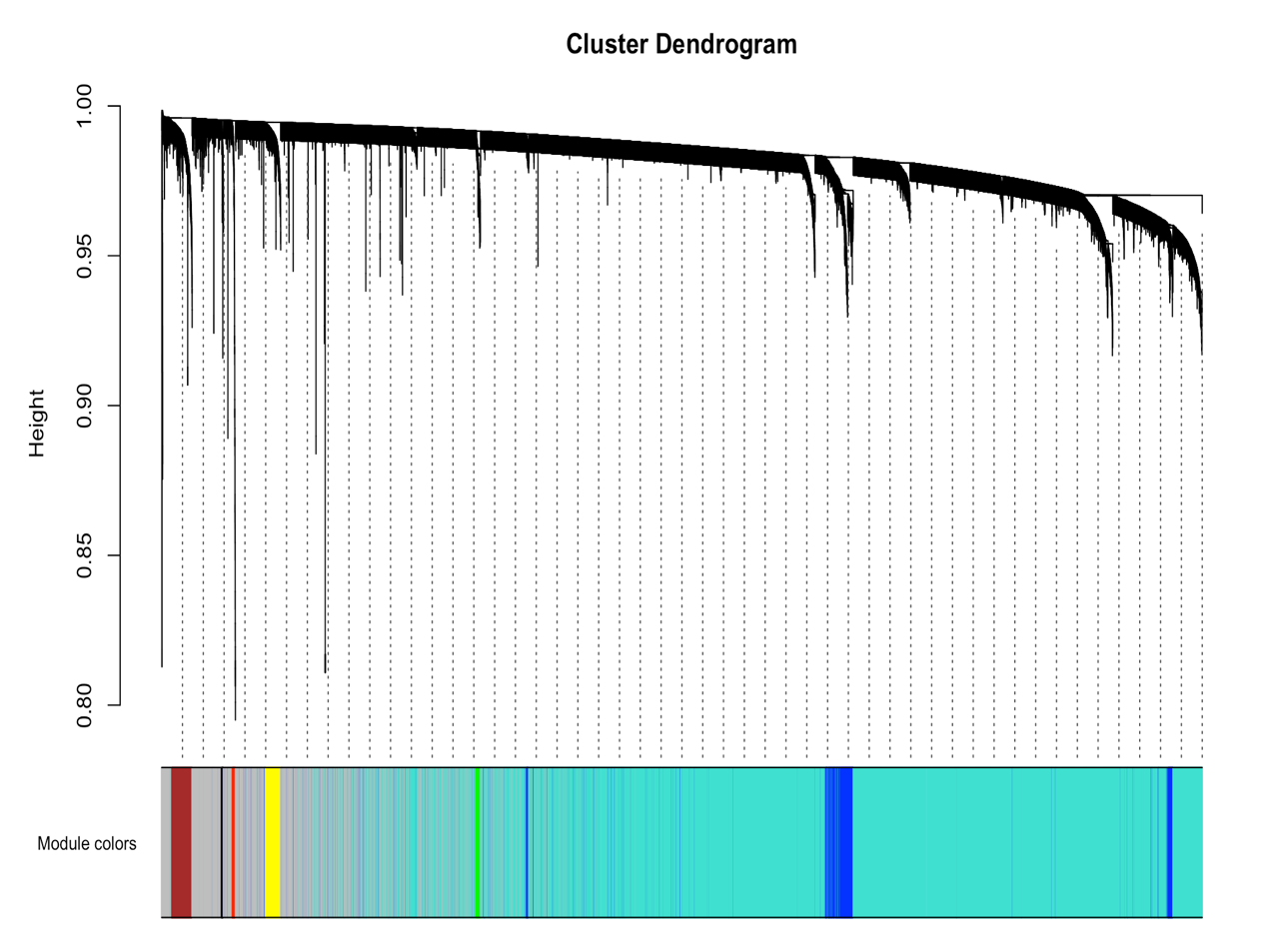


Supplementary Fig. S3. Dendrogram of all genes clustered based on a dissimilarity measure.


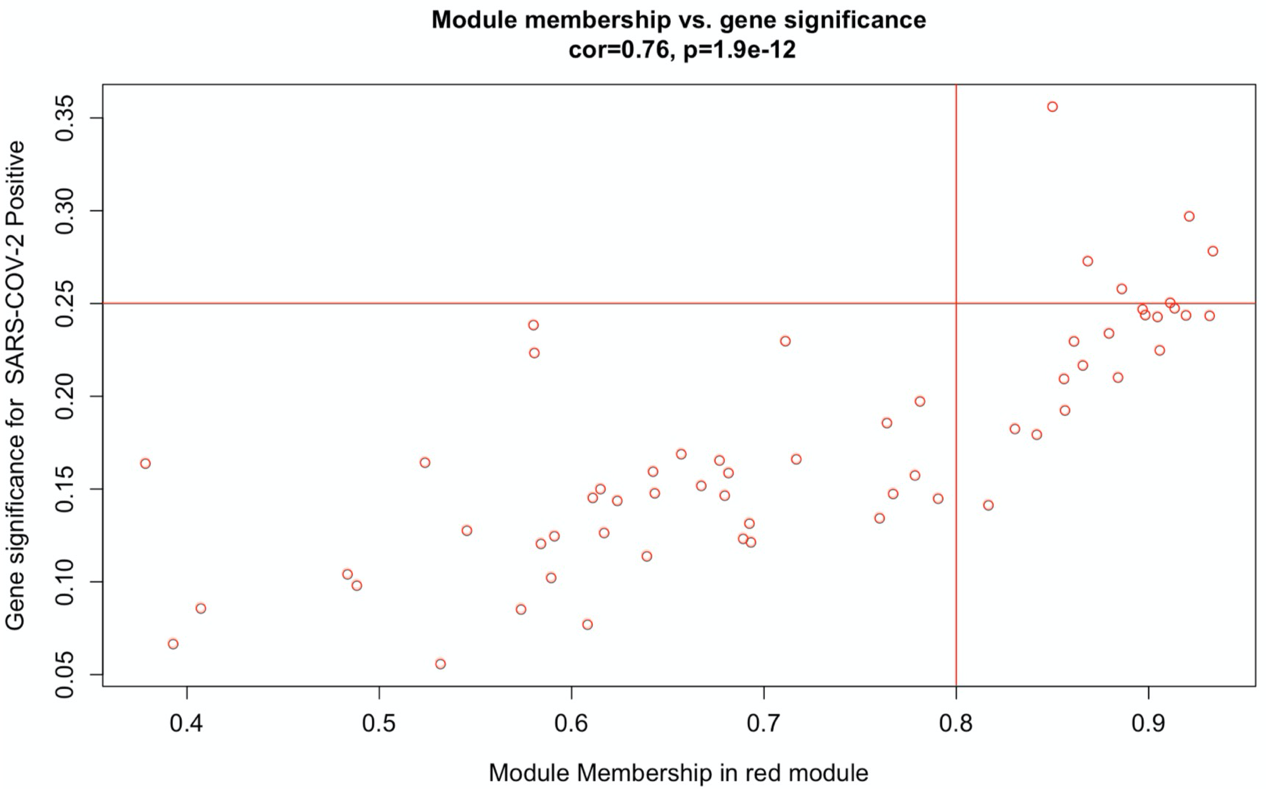


Supplementary Fig. S4. Scatter plot of module eigengenes (MEs) in red module. The red lines stand the selection thresholds for candidate hub genes.


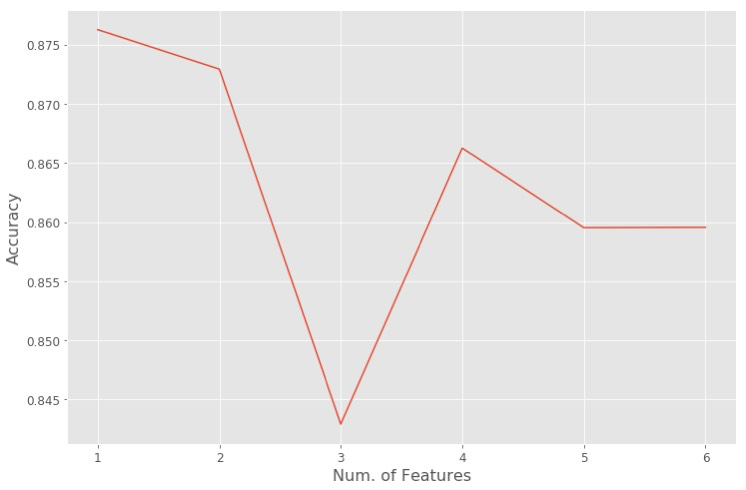


Supplementary Fig. S5. Classification accuracy versus number of genes, based on the combination of XGBoost and recursive feat. elimination with cross validation (RFECV).


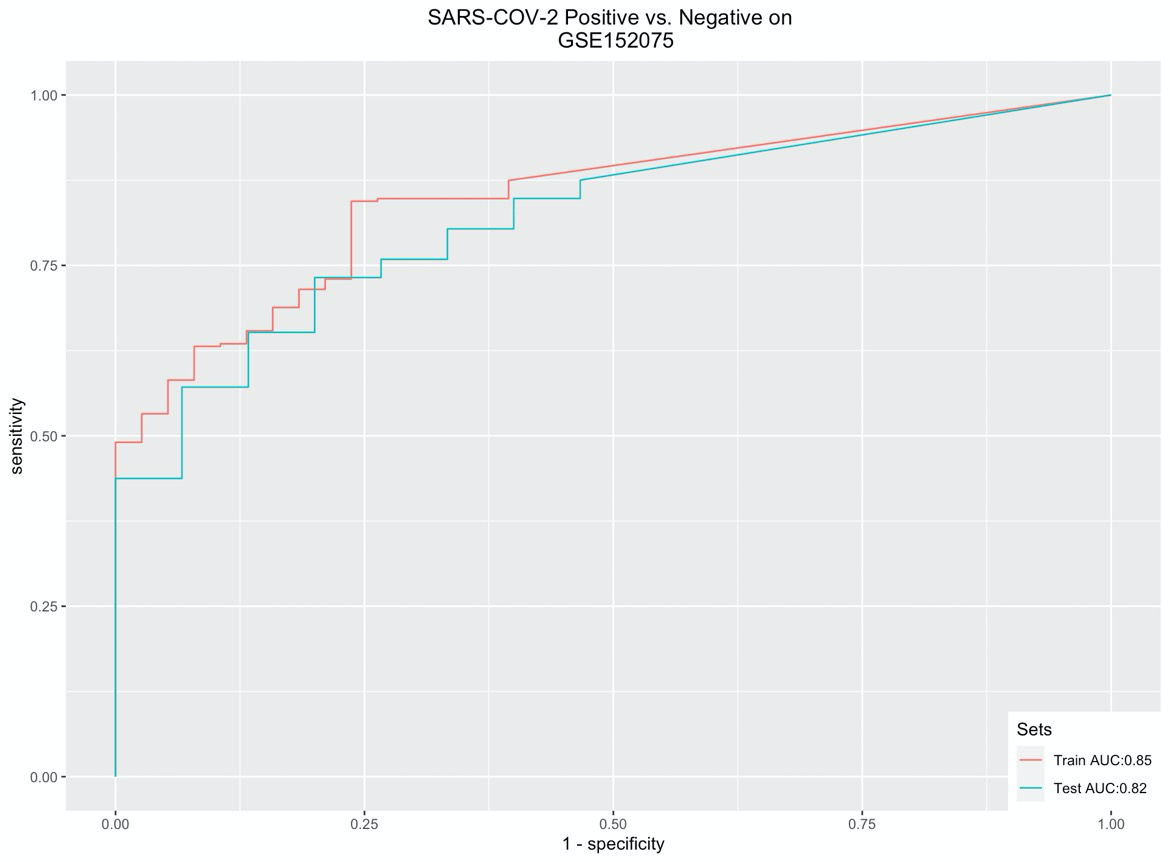


Supplementary Fig. S6. Evaluation of classification performance of the selected hub gene, phospholipase A2 group VII (PLA2G7), using dataset GSE152075. All the samples in GSE152075 were randomly assigned at a 7:3 ratio to a training set (299 samples) and a test set (129 samples).


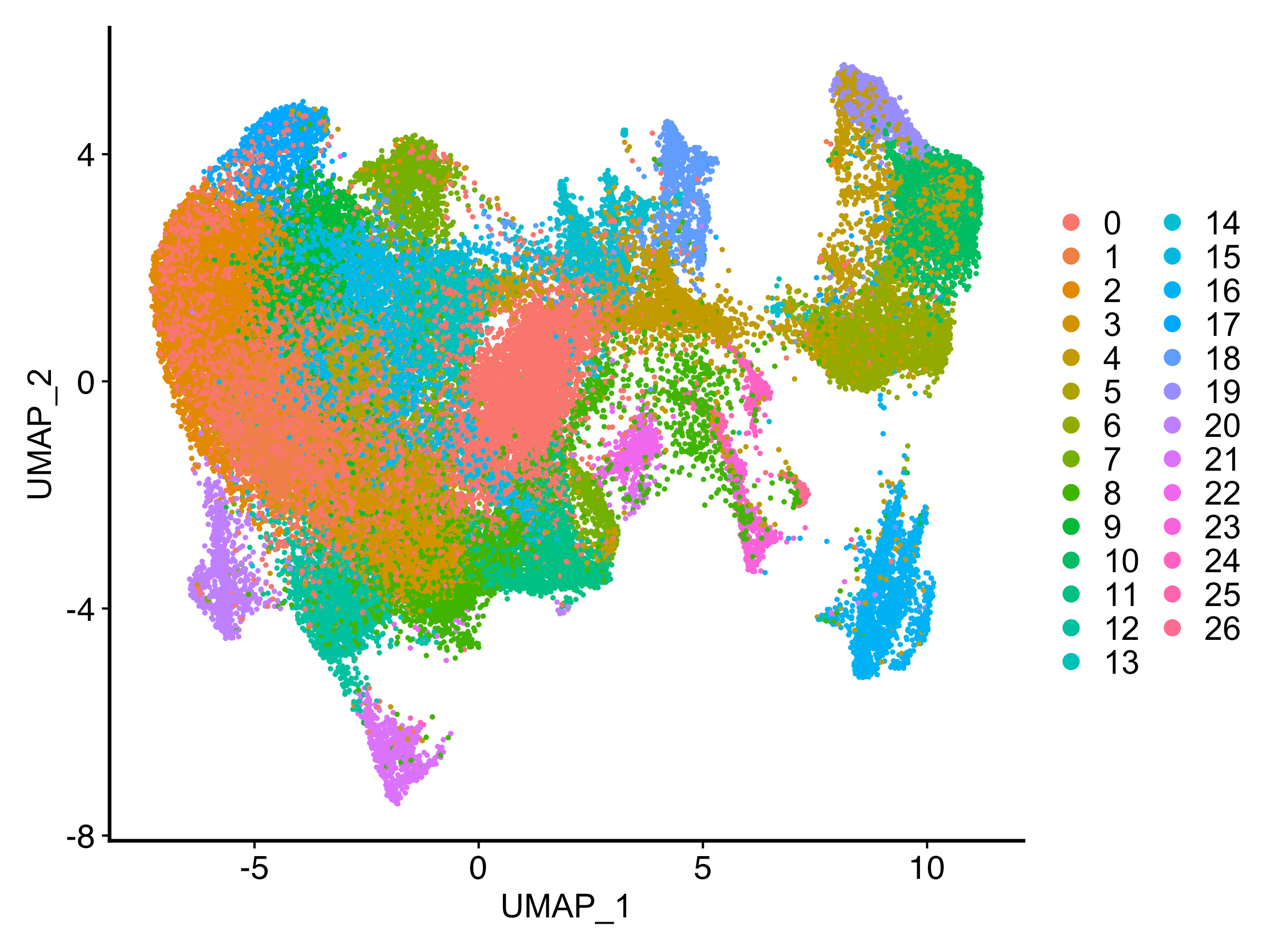


Supplementary Fig. S7. UMAP presentation of major clusters in bronchoalveolar lavage fluids (BALFs) from COVID-19 patients in GSE145926.

Supplementary Fig. S8. Expression of hallmark genes by different cell clusters. The markers and their corresponding cell clusters are listed on the right side.


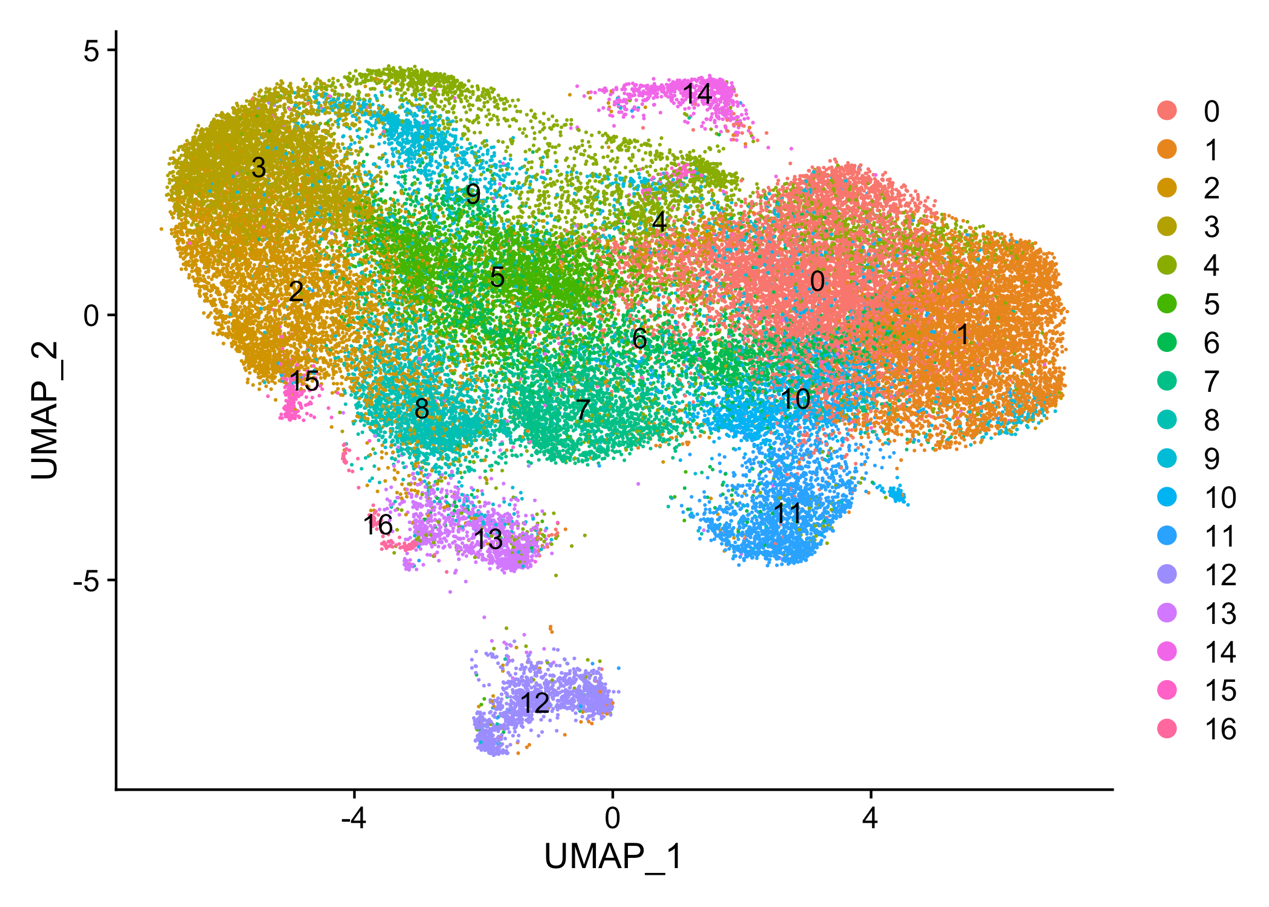


Supplementary Fig. S9 UMAP presentation of the major clusters of the re-integrated macrophages in BALFs from COVID-19 patients.

Supplementary Table 1 Genes in red module associated with SARS-COV-2 infection

| Ensembl_gene_id | Gene name | Gene_biotype |
| --- | --- | --- |
| ENSG00000225813 | AC009299.1 | processed_pseudogene |
| ENSG00000188778 | ADRB3 | protein_coding |
| ENSG00000229968 | AL022578.1 | processed_pseudogene |
| ENSG00000176134 | AL445665.1 | transcribed_unprocessed_pseudogene |
| ENSG00000274114 | ALOX15P1 | transcribed_unprocessed_pseudogene |
| ENSG00000198796 | ALPK2 | protein_coding |
| ENSG00000158874 | APOA2 | protein_coding |
| ENSG00000183092 | BEGAIN | protein_coding |
| ENSG00000235505 | CASP17P | transcribed_unprocessed_pseudogene |
| ENSG00000064205 | CCN5 | protein_coding |
| ENSG00000102245 | CD40LG | protein_coding |
| ENSG00000113361 | CDH6 | protein_coding |
| ENSG00000138395 | CDK15 | protein_coding |
| ENSG00000101938 | CHRDL1 | protein_coding |
| ENSG00000155833 | CYLC2 | protein_coding |
| ENSG00000260596 | DUX4 | protein_coding |
| ENSG00000225968 | ELFN1 | protein_coding |
| ENSG00000143340 | FAM163A | protein_coding |
| ENSG00000196666 | FAM180B | protein_coding |
| ENSG00000198173 | FAM47C | protein_coding |
| ENSG00000188859 | FAM78B | protein_coding |
| ENSG00000102755 | FLT1 | protein_coding |
| ENSG00000102287 | GABRE | protein_coding |
| ENSG00000145451 | GLRA3 | protein_coding |
| ENSG00000260062 | GOLGA2P11 | transcribed_unprocessed_pseudogene |
| ENSG00000196208 | GREB1 | protein_coding |
| ENSG00000152578 | GRIA4 | protein_coding |
| ENSG00000100433 | KCNK10 | protein_coding |
| ENSG00000075043 | KCNQ2 | protein_coding |
| ENSG00000183722 | LHFPL6 | protein_coding |
| ENSG00000244227 | LRRC77P | transcribed_unitary_pseudogene |
| ENSG00000130368 | MAS1 | protein_coding |
| ENSG00000149534 | MS4A2 | protein_coding |
| ENSG00000157064 | NMNAT2 | protein_coding |
| ENSG00000171246 | NPTX1 | protein_coding |
| ENSG00000112038 | OPRM1 | protein_coding |
| ENSG00000143867 | OSR1 | protein_coding |
| ENSG00000102109 | PCSK1N | protein_coding |
| ENSG00000172572 | PDE3A | protein_coding |
| ENSG00000146070 | PLA2G7 | protein_coding |
| ENSG00000074211 | PPP2R2C | protein_coding |
| ENSG00000259205 | PRKXP1 | processed_pseudogene |
| ENSG00000130032 | PRRG3 | protein_coding |
| ENSG00000232568 | RPL23AP35 | processed_pseudogene |
| ENSG00000137872 | SEMA6D | protein_coding |
| ENSG00000108387 | SEPTIN4 | protein_coding |
| ENSG00000112246 | SIM1 | protein_coding |
| ENSG00000115194 | SLC30A3 | protein_coding |
| ENSG00000185985 | SLITRK2 | protein_coding |
| ENSG00000228319 | SPATA2P1 | processed_pseudogene |
| ENSG00000139767 | SRRM4 | protein_coding |
| ENSG00000268447 | SSX2B | protein_coding |
| ENSG00000166317 | SYNPO2L | protein_coding |
| ENSG00000143858 | SYT2 | protein_coding |
| ENSG00000176358 | TAC4 | protein_coding |
| ENSG00000248320 | THAP12P9 | processed_pseudogene |
| ENSG00000146426 | TIAM2 | protein_coding |
| ENSG00000137462 | TLR2 | protein_coding |
| ENSG00000215296 | TMCO5B | transcribed_unitary_pseudogene |
| ENSG00000154764 | WNT7A | protein_coding |
